# Injectable and thermosensitive TGF-β1-loaded PCEC hydrogel system for *in vivo* cartilage repair

**DOI:** 10.1038/s41598-017-11322-w

**Published:** 2017-09-05

**Authors:** Tengfei Zhou, Xiaolong Li, Guo Li, Taoran Tian, Shiyu Lin, Sirong Shi, Jinfeng Liao, Xiaoxiao Cai, Yunfeng Lin

**Affiliations:** State Key Laboratory of Oral Diseases, National Clinical Research Center for Oral Diseases, West China Hospital of Stomatology, Sichuan University, Chengdu, China

## Abstract

Chondral defects pose a great challenge for clinicians to manage owing to the limited capacity for self-healing. Various traditional approaches have been adopted for the repair of these defects with unsatisfactory results. Cartilage tissue engineering techniques have emerged as promising strategies to enhance regeneration and overcome these traditional shortcomings. The cell-homing based technique is considered the most promising owing to its unique advantages. Thermosensitive hydrogels have been applied as scaffolds for biomedical applications with smart sol–gel response for altering environmental temperature. Transforming growth factor (TGF)-β1 is considered to be capable of promoting chondrogenesis. In this study, a novel TGF-β1-loaded poly(ε-caprolactone)–poly(ethylene glycol)–poly(ε-caprolactone) (PCEC) hydrogel was fabricated using simple procedures. Hydrogel characterization, rheological testing, component analysis, and assessment of sol–gel transition, *in vitro* degradation, and TGF-β1 release confirmed that this material possesses a porous microstructure with favorable injectability and sustained drug release. Full-thickness cartilage defects were induced on rat knees for *in vivo* cartilage repair for eight weeks. Micro-CT and histological evaluation provided further evidence of the optimal capacity of this novel hydrogel for cartilage regeneration with respect to that of other methods. Moreover, our results demonstrated that the cell-free hydrogel is thermosensitive, injectable, biodegradable, and capable of *in vivo* cartilage repair and possesses high potential and benefits for acellular cartilage tissue engineering and clinical application in the future.

## Introduction

Articular cartilage is a highly organized, hypocellular, and viscoelastic tissue. Cartilage defect, generally caused by trauma, tumor, or degenerative pathology, has long been proved to exhibit very limited intrinsic potential for self-healing^1–3^. Even a focal lesion may progress into serious degeneration such as arthritis, osteoarthritis, and necrosis, leading to chronic pain and functional impairment^4^. Researchers have ascribed this to the abnormal expression of proteinases such as matrix metalloproteinases (MMP), which degrade the chondral matrix constituents^5^. However, the clinical management of chondral defects is still considerably challenging. Traditional clinical therapies, including microfracture^6^, meniscal allografts^7^, arthroplasty^8^, and transplantation of perichondrium^9^ or chondrocytes^10–13^, have yielded some positive results but are subject to disadvantages such as procedure complexity, injuries, post-surgery complications, high cost, and immune concerns.

Thus, tissue engineering has become a promising strategy to overcome the above-mentioned drawbacks owing to its advantages, which include simplicity and the lack of a requirement for a second operation site^14–17^. Of these, the acellular or so-called cell-homing based technique, which achieves tissue repair by recruiting host cells and inducing specific cellular behaviors, has proved to be the most promising, with more simplified procedures, reduced time, and less concerns with respect the expensive official regulatory approval than conventional techniques^18–20^. Therefore, it has become an increasingly appealing tool for tissue repair over the past few years.

Smart hydrogels, water-swollen and physically or chemically crosslinked polymeric networks, have become the most sought-after scaffolds for biomedicine owing to their excellent biocompatibility, biodegradability, and “smart response” to environment stimuli such as temperature, pH, or chemicals^21–24^. In addition, the copolymer poly(ε-caprolactone)–poly(ethyleneglycol)–poly(ε-caprolactone), PCL–PEG–PCL (abbreviated as PCEC) displays thermosensitive behaviors typical of sol–gel transition under specific conditions^25–27^. At appropriate concentrations and molecular weight of the PEG and PCL segments, it could exist as a flowing solution at low temperatures and then transform into a solid gel-like substance at temperatures close to body temperature. This unique characteristic renders PCEC a promising injectable material for biomedical applications, particularly for *in situ* gelation tissue engineering^28–33^. The considerable syringeability enables the hydrogel solution to be applied via a minimally invasive surgery with ease, and it can be used to perfectly fill the crevices and corners of even an irregularly shaped cavity. Furthermore, the subsequent *in situ* gelation facilitates the fixing of the solution at the target locus, allowing it to function as a scaffold.

The applications of PCEC hydrogel have been broadly investigated in drug or biomolecule delivery, cell delivery^30^, anti-adhesion^34^, bactericide research^35^, and lately in tissue engineering^28–33^. Of late, researchers have primarily investigated its application in bone^32, 33^ and cartilage repair^30, 31^. Ji Sun Park examined its potential for cartilage repair in 2007, but the scaffold used in that study was composed of a PCL/PEG hydrogel and implanted with chondrocytes on nude mouse subcutaneous models^36^. Chao Yin Ko studied its application using a rabbit model of knee cartilage defects^30^. However, the cell-encapsulated PCEC hydrogel solution used could not gel spontaneously and required an extra photo-initiator to trigger the gelation process under 365 nm UV light. Thus, its clinical application potential may be limited by the need for application open sites to facilitate the subsequent necessary UV photo-crosslinking. Moreover, our previous study, conducted by Na Fu, has proved that PCEC can promote *in vivo* cartilage regeneration in rat knee models^31^. PCEC was subsequently fabricated into deposited films, which helped to resolve the material fixation problem but also led to difficulty in completely filling an irregular cavity. Despite tremendous efforts, we are still unable to achieve a comprehensive method that presents the advantages of minimal clinical access, defect cavity suitability, fast and viable fixation, local drug delivery, and capability for *in vivo* cartilage regeneration. Therefore, efforts to further investigate and understand the properties of this copolymer should proceed.

Transforming growth factor-β1 (TGF-β1) plays a significant role in various processes associated with chondrogenesis, such as chondrocyte growth and differentiation and enhancement of the biomechanical properties of neocartilage^37–39^. Local drug delivery systems (LDDS) have long been investigated owing to their potential for the transport of drugs or bioactive molecules to target loci and their sustained release, thereby overcoming the shortcomings of conventional methods by reducing adverse systemic reactions and enhancing therapeutic effects^40–44^. An optimal LDDS should release drugs sustainably, with its carrier being non-toxic, biodegradable, and biocompatible. LDDS of TGF-β1 have also been integrated with various carrier forms such as microspheres^45^, nanoparticles^46^, and scaffolds^47^ for sustained release.

In this study, we successfully fabricated an injectable thermosensitive TGF-β1-loaded PCEC hydrogel for cartilage repair for what is, to our knowledge, the first time. The sol–gel transition process was characterized and component analysis was also conducted. We further characterized *in vitro* hydrolytic degradation and evaluated TGF-β1 release quantitatively. Moreover, the acellular hydrogel solution was injected into surgically induced full-thickness cartilage defects in rat knees, and cell-homing based cartilage regeneration was evaluated at four and eight weeks post-surgery. The novel hydrogel system was favorably injectable and could gel spontaneously at body temperature without any external aid for crosslinking. The results of micro-CT and histological staining (Hematoxylin & Eosin [H&E] and Masson) indicated that cartilage regeneration was better in the TGF-β1-loaded group than in the control. We thus suggest that the new compound hydrogel proposed by us possesses advantages for clinical application and should further be studied for its future use in cartilage tissue engineering.

## Results

### Characterization of hydrogel morphology and properties

**Figure 1:**
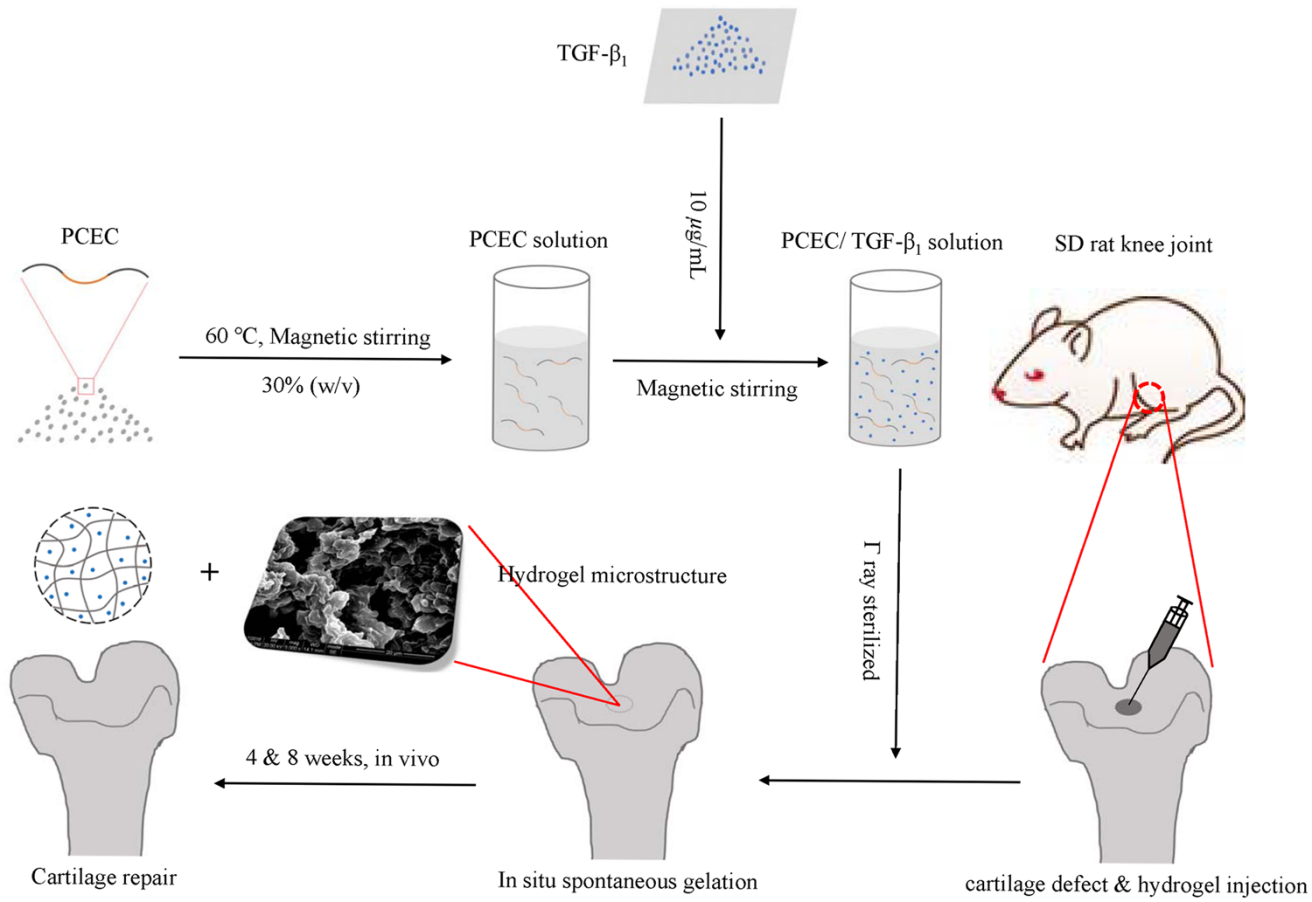
Schematic illustration of preparation process of TGF-β1/PCEC hydrogel system. Compound hydrogel was fabricated with simple procedures. Acellular hydrogels were implanted into rat knee joint defects via injection for *in vivo* cartilage regeneration at 4 and 8 weeks post-operation.

We successfully fabricated a new thermosensitive TGF-β1-loaded PCEC hydrogel with simple procedures. Schematic illustration of the preparation process, including relevant materials, solutions, resulting products, and *in vivo* cartilage repair of rat knee defects, are depicted in Fig. 1. Acellular hydrogels were applied via simple injection into knee defects for evaluation of *in situ* regeneration in *in vivo* cartilage after four and eight weeks.

**Figure 2:**
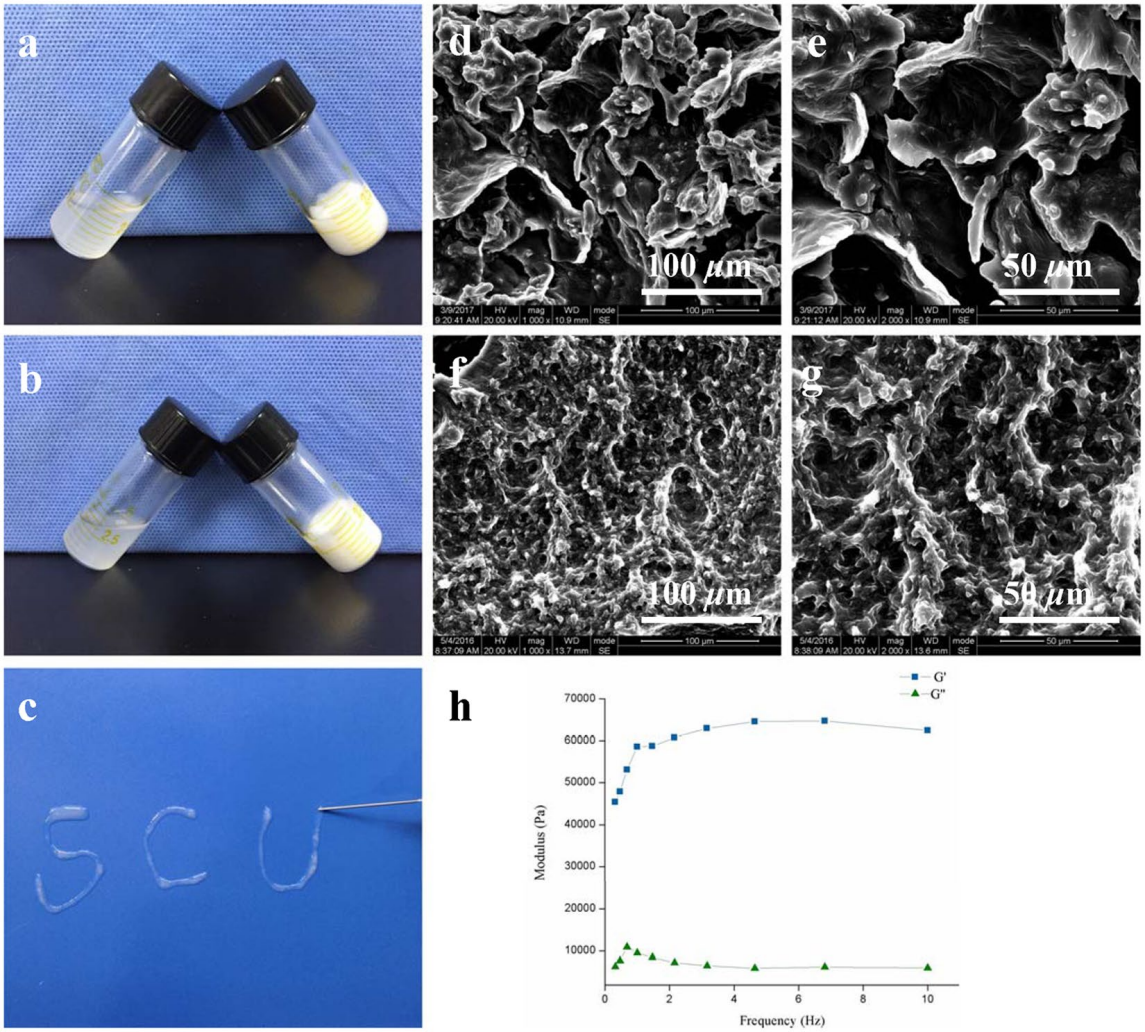
Characterization of hydrogel morphology and properties. (**a**) Sol-gel transition of bare PCEC hydrogel. (**b**) Sol-gel transition of TGF-β1 loaded PCEC hydrogel. (**c**) *In vitro* injectability test of TGF-β1 loaded PCEC hydrogel. (**d**,**e**) SEM characterization of bare PCEC hydrogel (scale bars: **d**, 100 *μ*m; **e**, 50 *μ*m). (**f**,**g**) SEM characterization of TGF-β1 loaded PCEC hydrogel (scale bars: **f**, 100 *μ*m; **g**, 50 *μ*m). (**h**) Rheology test of TGF-β1 loaded PCEC hydrogel (G’: storage modulus, G”: loss modulus).

The sol–gel transition of the hydrogel was assessed using the tube inverting test as shown in Fig. 2(a) and (b). The hydrogel solutions with or without TGF-β1 loaded were viscous and flowing, and both transformed into ivory gels within one minute after being heated to 37 °C. The liquid level when titled from the horizontal to inclined aspect, was observed to describe the sol–gel transition process.

The prepared TGF-β1-loaded hydrogel was favorably injectable at 25 °C with the solution easily passing through the 25-gauge needle, as shown in Fig. 2(c). In addition, rheology test revealed that the G′ value was significantly higher than the G′′ value in Fig. 2(h).

SEM images revealed that the hydrogel possessed obvious porous microstructure in Fig. 2(d) to (g). All hydrogel scaffolds were multilayered and resembled polygonal pores of micrometer grade. Moreover, a hierarchical pore structure could also be observed. TGF-β1-loaded hydrogel in Fig. 2(f) and (g); however, this appeared less porous and contained smaller pores.

### Component analysis of the hydrogel system

**Figure 3:**
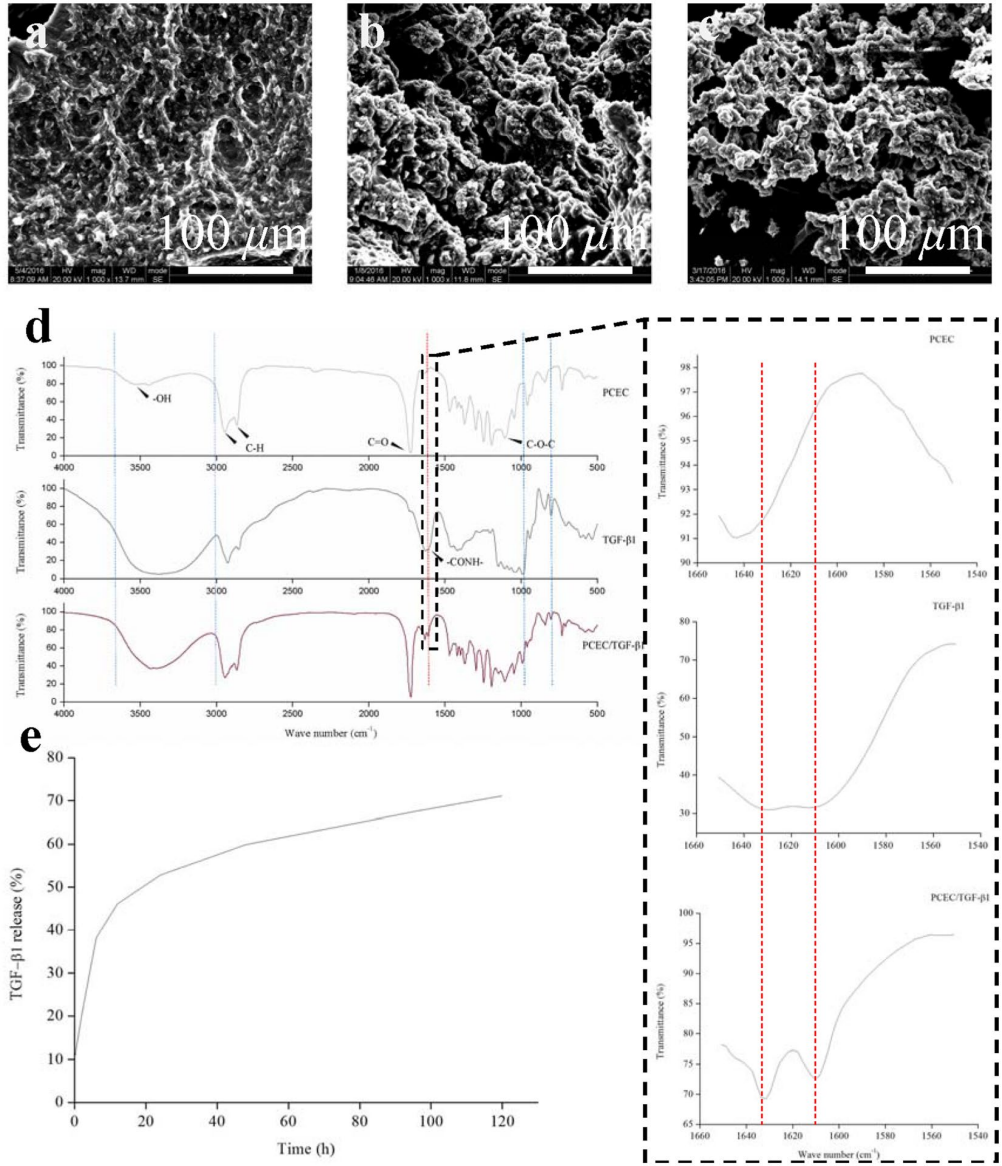
Hydrogel component analysis, *in vitro* degradation and TGF-β1 release. (**a**–**c**) SEM characterization of *in vitro* degradation of TGF-β1 loaded PCEC hydrogel at 0, 2, 4 weeks from the top downward respectively (scale bars: 100 *μ*m). (**d**) FTIR spectra analysis of bare PCEC hydrogel, TGF-β1 and TGF-β1 loaded PCEC hydrogel from the top downward respectively. New absorption peak at 1610 and 1630 cm^−1^, which referred to typical amide band I structure of TGF-β1 and couldn’t be detected in spectra of PCEC hydrogel, demonstrated the successful incorporation of TGF-β1 in hydrogel. (**e**) *In vitro* TGF-β1 release of compound hydrogel by ELISA test. Drug release came to gentle platform with a highest release rate of almost 70%.

As depicted in Fig. 3(d), FTIR infrared spectra (ATR mode) of bare PCEC hydrogel, TGF-β1, and TGF-β1-loaded PCEC hydrogel were analyzed starting from the top to the bottom. PCEC presented a typical absorption peak of hydroxyl groups (-OH) at around 3430 cm^−1^, 2926 cm^−1^, and 2854 cm^−1^ peaks, which could be attributed to -CH_2_- stretching vibrations of PCL units, and 1724 cm^−1^ for weak C = O vibration in PCL units and 1100 cm^−1^ because of the -COC- stretching of PEG units in -OCH_2_CH_2_- repeated units. TGF-β1 protein displayed the distinctive absorption peak of amide band I at 1610 and 1630 cm^−1^, which could be primarily assigned to the C = O bonding stretching vibration and partially to the N–H bonding. Furthermore, the TGF-β1-loaded PCEC hydrogel spectra highly resembled the PCEC spectra, as expected, but exhibited new absorption peaks at 1610 and 1630 cm^−1^, which could not be detected in PCEC, indicating the presence of TGF-β1 with its proteinic structure unimpaired.

### *In vitro* hydrolytic degradation behavior

The hydrolytic degradation test of hydrogels was conducted in enzyme-free PBS and characterized by SEM. Figure 3(a) to (c) depict the basipetal degradation of TGF-β1-loaded hydrogel at the first, second, and fourth week. The scaffold decomposed gradually with its integrity becoming impaired. Enlarged and increased cracks were observed on its surface.

### *In vitro* TGF-β1 release

*In vitro* TGF-β1 release properties were studied using a dynamic monitoring method and quantitatively evaluated by ELISA in Fig. 3(e). TGF-β1 released smoothly without any obvious burst because of the primary protection resulting from the incorporation of the hydrogel. It could be inferred that TGF-β1 released after the incorporating hydrogel decomposed gradually. Post 120 h, drug release reached a gentle halt, with about 30% of the drug contained inside the hydrogel.

### Macroscopic evaluation of cartilage repair

**Figure 4:**
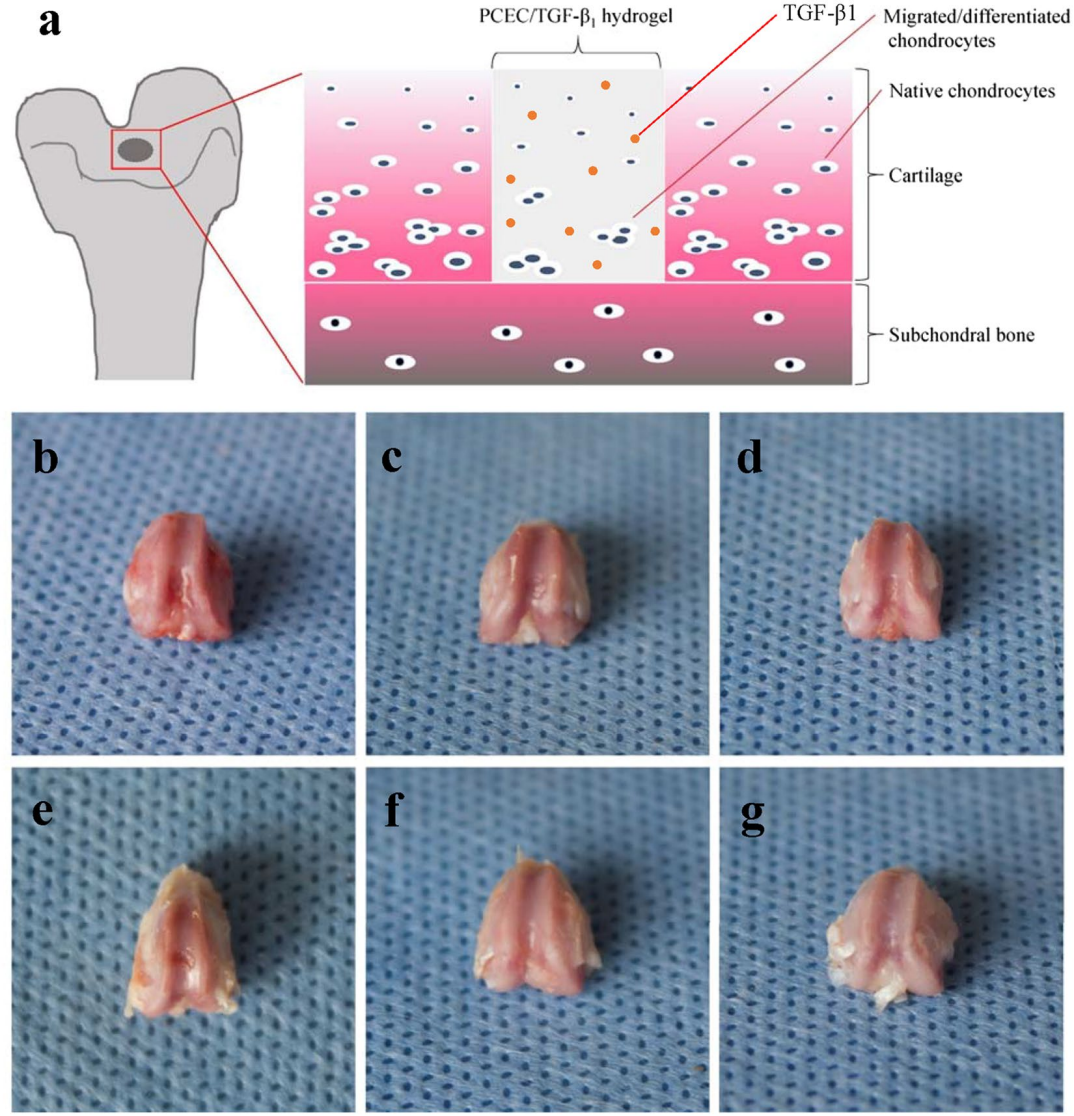
Hypothetical process of *in vivo* cartilage repair and macroscopic evaluation of knee joint samples. (**a**) Possible process of *in vivo* cartilage regeneration which we hypothesized. Repairing procedures may be mainly driven by migration and differentiation of bone marrow stem cells (BMSCs) from subchondral bone. TGF-β1 could play a promotive role during chondrogenesis. (**b**–**d**) Frontal views of rat knee joint defect samples at 4 weeks, with control, bare PCEC hydrogel and TGF-β1 loaded hydrogel groups arranged from left to right. Defects still caved in, with rough nascent tissue grown in. (**e**–**g**) Frontal views of rat knee joint defect samples at 8 weeks, with control, bare PCEC hydrogel and TGF-β1 loaded hydrogel groups arranged from left to right. Cartilage-like tissue filled in defects with gradually obscured boundaries, except for the control group.

Femur condyle samples at pre-determined time-points were harvested for gross evaluation of *in vivo* cartilage repair. Figure 4(a) depicts the possible cartilage regeneration approaches of our acellular hydrogel, which was hypothesized to associate with the migration of adjacent native chondrocytes, recruitment, and differentiation of stem cells such as bone marrow stem cells from subchondral bone, and the subsequent chondrogenesis promoted by TGF-β1.

Figure 4(b) to (d) refer to the control, hydrogel, and TGF-β1-loaded hydrogel group at four weeks and Fig. 4(e) to (g) for eight weeks from left to right, respectively. Compared with the four-week groups, all the eight-week samples displayed obviously larger coverage of the new tissue. In addition, the drug-loaded group exhibited better performance than the other groups in several respects: boundaries were gradually obscured with the content texture assembling the adjacent normal tissue; defect coverage also increased with the content shifting from soft nascent tissue to cartilage-like one; defects caved in with distinct steps surrounding the control group; and defects flattened gradually with more tissue growing. Moreover, in TGF-β1-loaded hydrogel group at eight weeks, defects had already been completely filled with high content of similar peri-native cartilage tissue, indicating optimal cartilage defect repair.

### Evaluation of reconstruction using micro-CT

**Figure 5:**
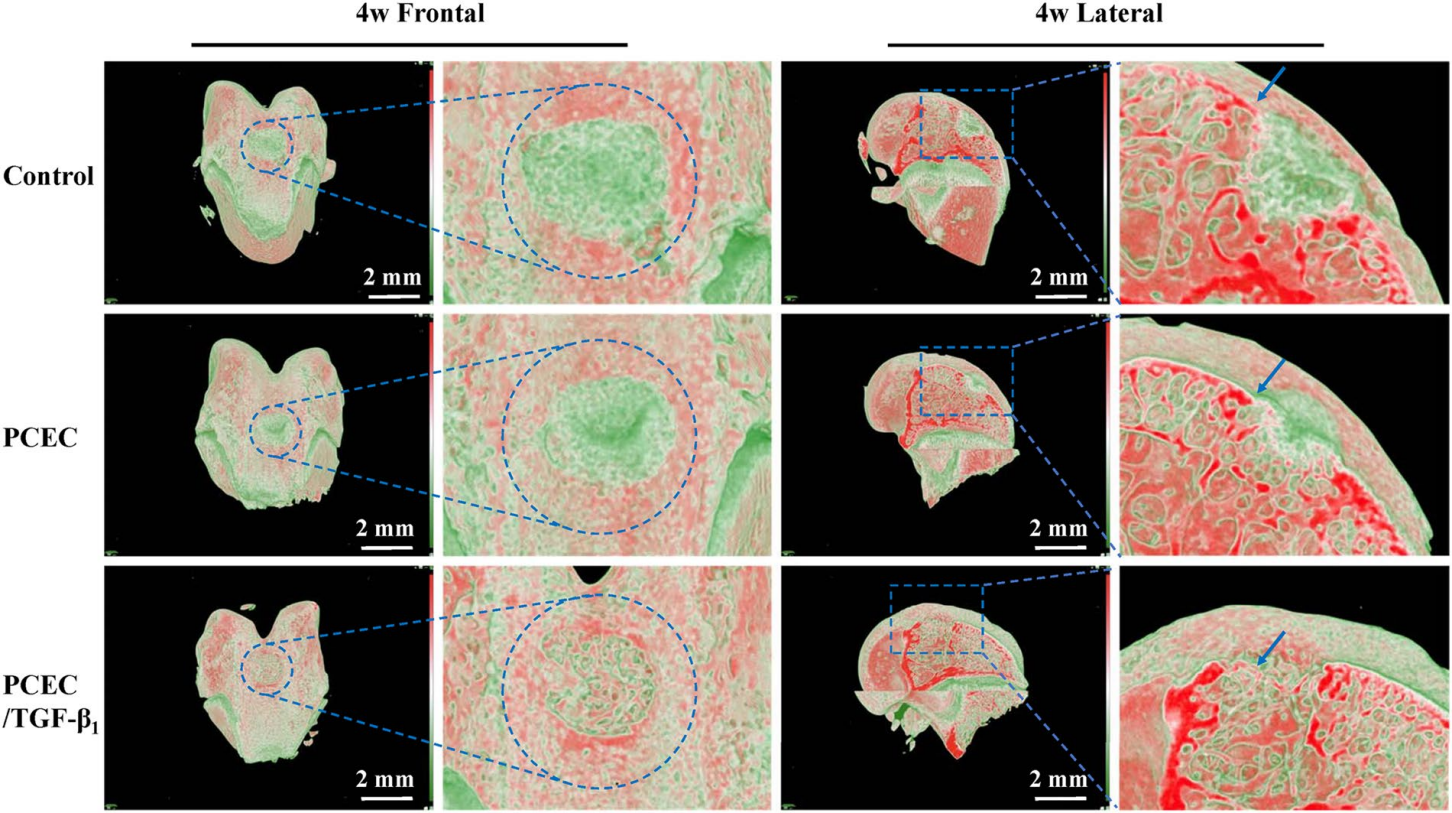
Micro-CT 3D reconstruction evaluation of knee joint samples at 4 weeks. frontal and lateral reconstructed images of the control, PCEC, TGF-β1 loaded PCEC group were displayed (scale bars: 2 mm). Blue circles indicated the surgical defect areas. Red areas symbolized hard or higher mineralized tissue such as bone and cartilage, green part represented low density ones like blank zone and blue arrow referred to the superficial cartilage layer. Blank zone were still obivious and TGF-β1 loaded hydrogel group possessed optimal repairing outcome. Frontal and lateral magnified images validated the regeneration process from surrounding margins and the bottom.

**Figure 6:**
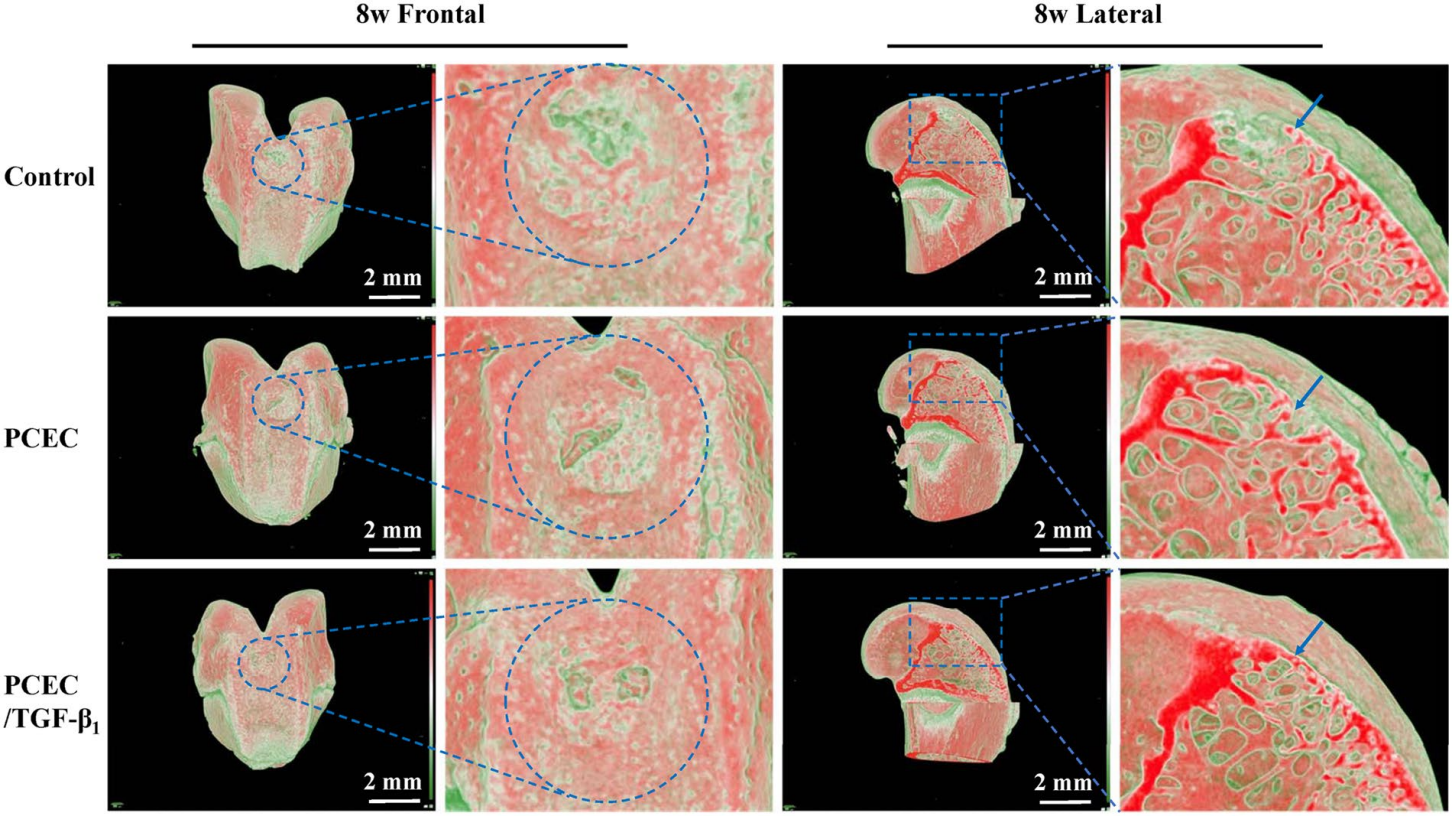
Micro-CT 3D reconstruction evaluation of knee joint samples at 8 weeks. frontal and lateral reconstructed images of the control, PCEC, TGF-β1 loaded PCEC group were displayed (scale bars: 2 mm). Blue circles indicated the surgical defect areas. Red areas symbolized hard or higher mineralized tissue such as bone and cartilage, green part represented low density ones like blank zone and blue arrow referred to the superficial cartilage layer. Blank zone were much smaller and TGF-β1 loaded hydrogel group possessed optimal repairing outcome. Frontal and lateral magnified images validated the regeneration process from surrounding margins and the bottom.

Knee joint samples were reconstructed into 3D images with blue circles and squares indicating their original and magnified defect areas, respectively. Frontal and lateral cutting view of four and eight weeks are shown in Fig. 5 and Fig. 6, respectively (scale bars: 2 mm). The 3D reconstructed images were exhibited with a red-green color mode, in which tissue density (Hounsfield unit, Hu) could be distinctly marked by red to green, with density from high to low. Red areas in figures represent high mineralized tissue-like bone and cartilage, and the deepest red strip in lateral cutting view refers to the superficial cartilage layer (blue arrows). Green areas indicate less-mineralized or unrepaired blank zones. It could be inferred from the frontal and lateral reconstructed images that neo-cartilage was regenerated from the margins and bottom of the defects toward the center and top. The newly formed hard tissue (red areas) gradually filled the defects over time and exhibited an increased tendency in the control, hydrogel, and drug-loaded hydrogel groups. At eight weeks post-surgery, defects in TGF-β1-loaded group were almost completely covered, denoted by red, in frontal view and fully filled in lateral view, with the cartilage layer being smooth and continuous (Fig. 6). However, the outcomes were not as defined for other groups, with still obvious blank zones remaining (green part in circles and squares) and a cracked cartilage layer. In summary, micro-CT results revealed our TGF-β1-loaded PCEC hydrogel possessed the optimal capacity of cartilage repair at eight weeks post-surgery compared to other groups.

### Histological evaluation

**Figure 7:**
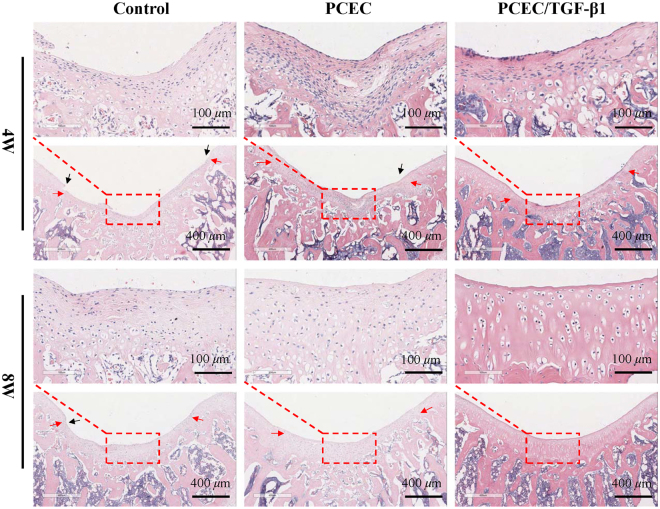
Hematoxylin-Eosin staining evaluation of *in vivo* cartilage regeneration in defects. Histomorphological evaluation of the control, PCEC, TGF-β1 loaded PCEC group were displayed at 4 and 8 weeks at 5x (scale bars: 400 *μ*m) and 20x (scale bars: 100 *μ*m) magnifications. hydrogels were biodegraded in all groups. new-formed tissue grew thicker with obscured demarcation and flattened steps, and gradually resembled host cartilage in tissue microstructure and cellular arrangement. TGF-β1 loaded hydrogel at 8 weeks possessed optimal repairing outcome, with defect completely replaced by typical cartilge-like tissue. (red arrows: tissue demarcation, black arrows: tissue steps).

Paraffin-embedded samples of control, hydrogel, and TGF-β1-loaded hydrogel groups were all sliced for H&E and Masson staining. The results of H&E staining are presented in Fig. 7. Hydrogels implemented could not be detected in any of the groups, indicating accelerated *in vivo* biodegradation. Coverage of the regenerated tissue was denser at eight weeks post-surgery with flattened tissue steps (black arrows). The demarcation (red arrows) between new and host cartilage, which could be distinguished by color and cellular morphology, was distinct at four weeks and was observed to move toward the center with obscured morphology in eight-week groups. Furthermore, it displayed effective formation of partial subchondral bone defects in the eight-week groups. Additionally, regional magnified images at 20× (in red rectangles) better exhibited tissue microstructure and cell types. At four weeks, the regenerated tissue was poorly organized with numerous fibroblast-like cells, denoting a basic fibrous repair process. In contrast to the control and bare hydrogel group, the TGF-β1-loaded hydrogel group manifested with decreased fibroblasts, more chondrocyte-like cells proliferating from the bottom, and more extra cellular matrix (ECM) deposition, suggesting primary cartilage repair. Furthermore, eight-week groups exhibited a cartilage-like morphology with an increased tendency of a homogenous tissue structure. At eight weeks in the TGF-β1 group, defects had been completely repaired, with highly organized cartilage-like tissue observed that was similar to adjacent host cartilage. No defect steps remained and a continuous smooth surface was obtained with typical chondrocytes arranged in columns.

**Figure 8:**
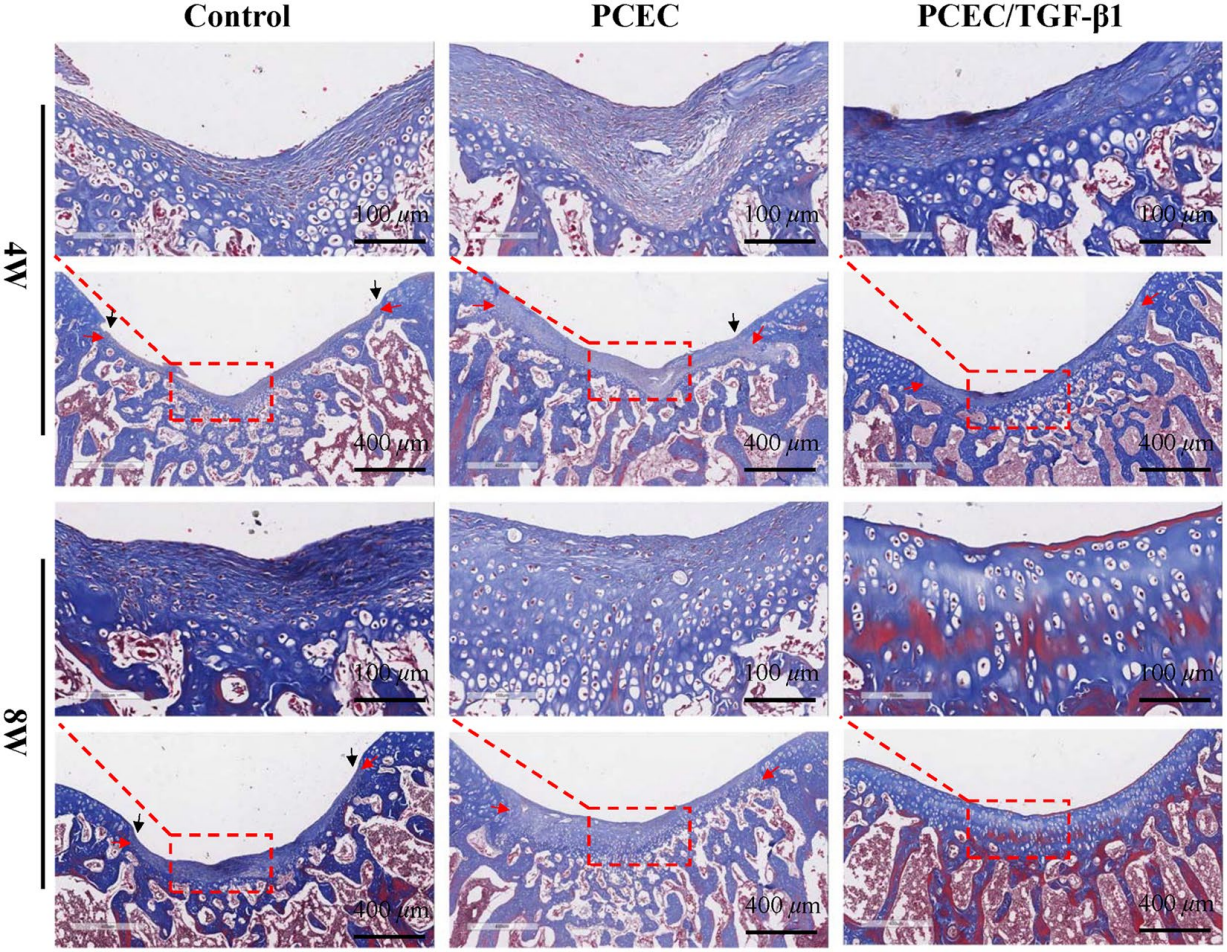
Masson staining evaluation of *in vivo* cartilage regeneration in defects. Evaluation of tissue histomorphology and deposition of cartilage matrix of the control, PCEC, TGF-β1 loaded PCEC group were displayed at 4 and 8 weeks at 5x (scale bars: 400 *μ*m) and 20x (scale bars: 100 *μ*m) magnifications. Hydrogels were biodegraded in all groups. Regional magnified images of 4 and 8-weeks revealed the process transformation from basic fibrous tissue into cartilage-like one. In TGF-β1 loaded hydrogel at 8 weeks, defects was almost fully repaired with smooth surface, homogenous matrix depostion and typical chondrocyte arrangement. (red arrows: tissue demarcation, black arrows: tissue steps).

Masson staining clearly demonstrated the progressive transformation of *in vivo* cartilage repair from basic fibrous tissue to cartilage-like tissue (Fig. 8), which was validated by H&E staining. In four-week groups, defects were filled with numerous fibroblasts when observed with 20× vmagnification. In contrast, defects at eight weeks presented with more cartilage-like tissue proliferating from the bottom and decreased fibrous contents. ECM was stained homogeneously with increasing similarity to peri-native cartilage. Additionally, defects of TGF-β1 group at eight weeks displayed a typical hyaline cartilage structure with cells encapsulated in lacunas with columnar arrangement.

## Discussion

To the best of our knowledge, it was the first thermosensitive TGF-β1 loaded PCEC hydrogel fabricated with facile procedures for *in vivo* cartilage repair on rat knee joints. Sol-gel transition assessment revealed its desirable thermosensitive property, which kept hydrogel flowing at low temperature and gel-like at around body temperature. *In vitro* injectability test further proved that. Rheology test has been broadly employed for evaluation of hydrogel viscoelastic properties, with storage modulus representing the elasticity part and loss modulus referring to the viscosity part of hydrogel^26^. With G’ higher than G” value, it could be confirmed that our TGF-β1 loaded hydrogel stayed at gel phase at 37 °C. Our results were in good consistence with previous work^26, 48^. Smart thermosensitivity enabled hydrogel to be applied as an injectable biomaterial, with virtues of minimal invasion and *in situ* gelation as a primary scaffold for tissue regeneration.

Porous microstructure provided our hydrogel with desirable microenvironment for cells like chondrocytes and bone marrow stem cells (BMSCs) to attach to, grow in and proliferate. Moreover, its hierarchical pores, comprising interconnective macro-pores with canals and micro-pores on their walls, may help to promote nutrients supply and removal of cellular metabolic waste of scaffolds^49^.

Effects of molecular weight combinations of PCL-PEG-PCL segments and concentration of PCEC copolymer on the sol-gel transition properties have been broadly investigated^48, 50^. Moreover, it was proved that PCEC, with a molecular weight of 1,000–1,000–1,000 for PCL-PEG-PCL blocks respectively and a copolymer concentration of 30%, had a gel window of 20–50 °C^50^. Our new hydrogel system was prepared with same formulations accordingly, thus it was supposed to maintain a flowing state at low temperature and gel at body temperature.

FTIR-ATR has been commonly employed for assessing components and microstructural properties of various hydrogels^32^. Thus, in this study, it was adopted to accurately verify the detailed components existence. TGF-β1 was proved to be successfully incorporated into our hydrogel system with its characteristic amide bond unimpaired.

*In vitro* degradation tests of PCEC hydrogel have been reported in hydrolytic or enzymatic ways, but most of them focused on the weight loss with less concerns about the morphological changes during degradation^26, 32, 50^. And PCEC hydrogel of 20% was reported to degrade within three weeks *in vivo* in subcutaneous models^50^. our results were still in compliance since *in vivo* degradation was an intricate enzyme-driven process which was generally considered to go on with accelerated speed^26^.

PCEC hydrogel has been employed to delivery various drugs such as hydrophobic, hydrophilic and protein ones owing to its unique amphiphilic micelle-like structure^40–43^. Additionally, researches have proved the protection and bioactivity preservation of PCEC hydrogel analogies to proteins like insulin^43^. Our release results may still seem fast and we ascribed this to the insufficiency of hydrogel protection. Drugs incorporated into nanoparticles have been proved with more sustained release due to the dual incorporation of hydrogel particles and external matrix, which also suggested us the direction for its further improvement. Anyway, our TGF-β1 loaded PCEC hydrogel was still a sustainable local drug delivery system (LDDS) with a virtue of loading simplicity.

H&E staining was generally conducted for evaluation of tissue morphology and the interface between native and new-formed tissue^31, 47^. Masson staining was also favored as special staining test due to its enhanced exhibition quality of tissue microstructure and matrix component like collagenous fibers^51^. In summary, our micro-CT and histological results revealed that, compared with its opponents, TGF-β1 loaded hydrogel was more effective for *in vivo* cartilage repair with hyaline cartilage-like tissue regenerated. Besides, the process typically developed from basic fibrous tissue, fibrous/chondral mixtures to the final hyaline cartilage-like tissue, which was in consistence with most current researches^31, 47, 51^. Chondrocytes were considered as terminally differentiated cells which could barely proliferate^52^. Thus, we hypothesized that the regeneration was mainly triggered by the recruitment of stem cells such as bone marrow stem cells (BMSCs) from subchondral bone, adipose-derived stem cell (ASCs) from adipose tissue below or other stem cell types from surrounding synovium. And TGF-β1 released form hydrogel scaffold facilitated the chondrogenesis by promoting chondrocytes growth and differentiation. Migration and matrix secretion of native chondrocytes from adjacent cartilage also partially helped this process.

Chao Yin Ko has explored the *in vivo* cartilage repair of PCEC hydrogel on rabbit knee joint models^30^. However, his system encapsulated chondrocytes inside hydrogel, which was not based on acellular cell-homing since these exotic cells may have played a great role in the regeneration process. Furthermore, his hydrogel solution failed to gel spontaneously and demanded extra photo-initiator inside to trigger its gelation process under 365 nm UV. Thus, its clinical application potential may be impaired since it was limited to open sites for an indispensable UV photo-crosslinking subsequently. We encapsulated TGF-β1 into the acellular thermosensitive PCEC hydrogel and by combining their advantages together, thus we may improve its regeneration capacity and broaden its clinical application field. Evaluation for biomechanical properties and long-term follow-up study of regenerated cartilage should be considered in the future to confirm its repair quality without unfavorable deterioration and degeneration. Thus, we shall still endeavor to further investigate and understand this interesting copolymer.

## Conclusion

In this study, a novel injectable TGF-β1-loaded PCEC hydrogel was fabricated with simple procedures. The hydrogel scaffold was characterized as having a porous microstructure, excellent injectability, and thermosensitivity. System component, *in vitro* hydrolytic degradation, and TGF-β1 release were further evaluated. Full-thickness cartilage defects were performed on rat knee joints for cell-free hydrogel implantation by injection. Micro-CT and histological evaluation confirmed the optimal *in vivo* repair outcomes of TGF-β1-loaded hydrogel with hyaline cartilage-like tissue regeneration at eight weeks. Therefore, we suggest that this novel injectable and thermosensitive acellular hydrogel is promising in the field of cell-homing based osteochondral tissue engineering and also affords great benefits and potential for clinical cartilage repair.

## Methods

### Materials

Copolymer PCEC (molecular weight: 1000–1000–1000 for PCL–PEG–PCL segments, respectively) was provided by State Key Laboratory of Biotherapy and Cancer Center (Sichuan University, Chengdu, China). TGF-β1, ELISA kits (BioSource international Inc., Camarillo, CA) for the release test, EDTA, paraffin, and all staining reagents were purchased from Sigma-Aldrich. Other analytical-grade chemical reagents and solvents were all obtained from the State Key Laboratory of Oral Diseases, National Clinical Research Center for Oral Diseases (West China Hospital of Stomatology, Sichuan University, Chengdu, China) and were used as received.

### Fabrication of TGF-β1-loaded PCEC hydrogel

PCEC (1.5 g) was dissolved by magnetic stirring in 5 mL of double distilled water for 2 h at approximately 60 °C to obtain a hydrogel solution with a concentration of 30% (w/v). TGF-β1-loaded hydrogel solution was prepared by dissolving 50 μg TGF-β1 in the aforementioned solution after it had cooled to room temperature to achieve a concentration of 10 μg/mL. The final TGF-β1-loaded PCEC hydrogel solution was then mixed homogeneously by means of magnetic stirring for 30 min and stored at 4 °C until further use. The prepared hydrogel solutions were all sterilized with γ-ray for 24 h before *in vivo* cartilage repair.

### Sol–gel transition test

The tube inverting test is commonly employed to characterize the sol–gel transition process of thermosensitive hydrogels. In this study, hydrogels with or without TGF-β1 loaded inside were poured into transparent vials and then slowly heated to 37 °C in a water bath. Vials were inverted or tilted to a 45° angle after maintaining them at 37 °C for a period of time. Sol–gel transition was evaluated by observing the level of the hydrogel liquid to determine whether it tilted. Hydrogel state (viscous or gel-like) and transition time was also analyzed and recorded (n = 2).

### Evaluation of the injectability of the formulation

Syringe with a 25-gauge microfine needle was filled with TGF-β1-loaded PCEC hydrogel solution, and the injectability of formulations was characterized by passing the solution through the needle at 25 °C.

### Rheology test

An R/S plus Mo8-219 rheometer (Brookfield, Middleboro, USA) was used to quantitatively evaluate the rheological behavior of TGF-β1-loaded PCEC hydrogel by dynamically monitoring variations of hydrogel storage modulus (G′) and loss modulus (G″) as a function of frequency (Hz). TGF-β1-loaded hydrogel was spread onto a parallel plate (diameter: 40 mm) for testing in accordance with standard practiceD, and the concerned parameters were evaluated at 37 °C. Tests were performed in triplicate (n = 3).

### Scanning electron microscopy (SEM) characterization

Samples were freeze-dried for 48 h to eliminate water disturbance in SEM and were then separated into small mats. SEM (HITACHI S-4800, TOKYO, Japan) was used to characterize hydrogels at an accelerating voltage of 20 kV. After being mounted on aluminum stubs, samples were sputter-coated with a layer of gold powder for testing for 1.5 min at 15 mA. The microstructure of the hydrogel with or without TGF-β1 (n = 2), including pore shape, pore size, and pore interconnectivity, was characterized and evaluated.

### Component analysis

Attenuated total reflection Fourier transform infrared spectroscopy (FTIR-ATR) was conducted using a Nicolet 5700 FTIR spectrometer (Thermo Electron Corp, Madison, WI) equipped with the accessory Nicolet Smart Orbit ATR to further characterize the fine components of our hydrogel system. Samples were freeze-dried for 48 h and about 1 mg of them was used for tests; infrared spectra of TGF-β1, bare PCEC hydrogel, and TGF-β1-loaded PCEC hydrogel were measured using the KBr disk method (n = 3). Finally, the Origin Pro 2016 software was employed to depict the spectra.

### Characterization of *in vitro* hydrolytic degradation

Hydrogel *in vitro* degradation test was conducted in enzyme-free phosphate-buffered saline (PBS; 0.01 M, pH 7.4), and hydrogel morphological changes during degradation were characterized by SEM. Samples of TGF-β1-loaded PCEC hydrogel were shaped into similar mats and incubated in 5 mL PBS for four weeks (n = 4). The entire process was performed at 37 °C in a shaking water bath. PBS medium was refreshed every 7 d during incubation. Degraded samples were collected in the second and fourth weeks, carefully washed, and freeze-dried for 48 h before observation using SEM.

### In vitro TGF-β1 release

We quantitatively defined the *in vitro* TGF-β1 release property of this compound hydrogel system in enzyme-free PBS via a dynamic monitoring method. In brief, samples of 20 mg PCEC hydrogel with TGF-β1 loaded were immersed into 1 mL 1× PBS in sterile tubes and were then incubated in a shaking water bath at 135 rpm at 37 °C (n = 3). The time intervals were pre-determined at 0, 2, 6, 12, 24, 48, 96, and 120 h after incubation. The hydrogel suspension was centrifuged, and the entirety of the supernatant was collected at each pre-determined time point for the drug release test. An ELISA kit (BioSource International Inc., Camarillo, CA) was then used to measure the concentrations of TGF-β1 released into the supernatant.

### *In vivo* animal model of cartilage defect

The animal experimental protocol was reviewed and approved by our institutional review board (IRB), and experimental procedures were in compliance with the relevant laws and ethical principles.

Eighteen healthy specific pathogen free (SPF)-grade SD rats from Sichuan University Animal Center, aged about 12 weeks and weighing 280 g on an average, were used in this study. All animals were anaesthetized by chloral hydrate through intraperitoneal injection at 0.3–0.35 mL/100 g. Bilateral knee joints were exposed by lateral dislocation of the patella after a medial parapatellar incision. Full-thickness cartilage defects were surgically created at the femoropatellar groove on both hind leg knee joints, which penetrated the cartilage layer into the superficial part of the subchondral bone. All defects were uniformly created via holes with a depth of 1.5 mm and a diameter of 2 mm using an electric drill.

### *In vivo* hydrogel injection

All hydrogel materials used in this study were exposed to γ-rays for 24 h beforehand for disinfection without impairing the bioactivity of TGF-β1 and were then stored at 4 °C. Eighteen SD rats were adopted with 36 knee defect samples, of which 12 defects were subjected to the TGF-β1-loaded PCEC hydrogel and 12 to the bare PCEC hydrogel; 12 defects comprised the blank control group. Hydrogel samples were all maintained at room temperature for a period of time and were smoothly injected into defects for 4.5 μL per defect immediately after the cartilage defects were created. About 1.5 minutes later, solution gelated and knee joint synovium, muscles, and skin were carefully sutured for closure. The rats were all in good condition and did not present limping, and had *ad libitum* access to food and water post-surgery.

### Macroscopic evaluation of cartilage repair

After four and eight weeks post-surgery, rats were sacrificed via an overdose of the anesthetic, and bilateral distal parts of femur condyles were harvested for further evaluation (18 samples for each time and six samples for each group of a time-point). Samples were all carefully dissected without excess soft tissue and fixed in 4% paraformaldehyde for 48 h. Frontal photographs of knee joints were captured for gross evaluation of *in vivo* cartilage repair of defect sites.

### Evaluation of reconstruction via micro-CT

All samples were fixed in 4% formaldehyde for 48 h after removing excessive soft tissue and were set immobile inside the scanning tubes with their femoral axis perpendicular to the scanning plane. Micro-CT scanning (Y. Cheetah, YXLON International GmbH, Germany) was then performed to reconstruct images of the microstructural regeneration of cartilage and subchondral bone in the defect areas. Scanning process was conducted with the following parameters: voltage, 56 kV; current, 61 μA; and voxel resolution, 0.012 mm. All images were subsequently reconstructed with the VGS Studio Max software to generate three-dimensional structures. Frontal and lateral cutting views were used to evaluate the regenerated cartilage and subchondral bone.

### Histological evaluation

Samples from the control, hydrogel, and TGF-β1-loaded hydrogel group were decalcified using 15% ethylenediaminetetraacetate (EDTA)-buffered saline solution for 15 days, dehydrated with gradient ethanol solutions, and then embedded in paraffin blocks. Next, they were then sectioned into 5-μm-thick slices perpendicular to the longitudinal axis, and stained with H&E for general tissue histomorphology analysis and with Masson staining for better structural assessment of cartilage matrix composition and deposition. All stained slices were scanned with tissue scanner equipment (Aperio, ScanScope XT, USA) and analyzed using a software (Aperio, Image Scope, USA) under magnifications of 5× and 20×.

### Statistical analysis

Student’s t-test and one-way analysis of variance (ANOVA) were performed for statistical analysis in this study, with all data evaluated by SPSS 20.0 software (SPSS, Chicago, IL, USA), and the statistical significance level was set at *P* < 0.05.

## References

[1] Findlay DM, Kuliwaba JS (2016). Bone-cartilage crosstalk: a conversation for understanding osteoarthritis. Bone Res..

[2] Lacy KW, Cracchiolo A, Yu S, Goitz H (2016). Medial Femoral Condyle Cartilage Defect Biomechanics: Effect of Obesity, Defect Size, and Cartilage Thickness. Am. J. Sports Med..

[3] Kitamura N, Yokota M, Kurokawa T, Gong JP, Yasuda K (2016). *In vivo* cartilage regeneration induced by a double-network hydrogel: Evaluation of a novel therapeutic strategy for femoral articular cartilage defects in a sheep model. J. Biomed. Mater. Res. A.

[4] Chang NJ (2015). The repair of full-thickness articular cartilage defect using intra-articular administration of N-acetyl-D-glucosamine in the rabbit knee: randomized controlled trial. Biomed. Eng. Online.

[5] Grimaud E, Heymann D, Redini F (2002). Recent advances in TGF-beta effects on chondrocyte metabolism. Potential therapeutic roles of TGF-beta in cartilage disorders. Cytokine Growth Factor Rev..

[6] Mithoefer K, McAdams T, Williams RJ, Kreuz PC, Mandelbaum BR (2009). Clinical efficacy of the microfracture technique for articular cartilage repair in the knee: an evidence-based systematic analysis. Am. J. Sports Med..

[7] Harris JD, Cavo M, Brophy R, Siston R, Flanigan D (2011). Biological knee reconstruction: a systematic review of combined meniscal allograft transplantation and cartilage repair or restoration. Arthroscopy.

[8] Matsunaga D, Akizuki S, Takizawa T, Yamazaki I, Kuraishi J (2007). Repair of articular cartilage and clinical outcome after osteotomy with microfracture or abrasion arthroplasty for medial gonarthrosis. Knee.

[9] Bruns J, Meyer-Pannwitt U, Silbermann M (1992). The rib perichondrium. An anatomical study in sheep of a tissue used as transplant in the treatment of hyaline-cartilage defects. Acta Anat (Basel)..

[10] Perka C (2000). Joint cartilage repair with transplantation of embryonic chondrocytes embedded in collagen-fibrin matrices. Clin Exp Rheumatol.

[11] Yu FY (2010). Mechanisms of autologous chondrocytes mass transplantation in the repair of cartilage defects of rabbits’ knee. Zhongguo Gu Shang.

[12] Horas U, Pelinkovic D, Herr G, Aigner T, Schnettler R (2003). Autologous chondrocyte implantation and osteochondral cylinder transplantation in cartilage repair of the knee joint. A prospective, comparative trial. J. Bone Joint Surg. Am..

[13] Lin L (2014). Sonic hedgehog improves redifferentiation of dedifferentiated chondrocytes for articular cartilage repair. PLoS One.

[14] Shao X (2017). Tetrahedral DNA Nanostructure: A Potential Promoter for Cartilage Tissue Regeneration via Regulating Chondrocyte Phenotype and Proliferation. Small.

[15] Liao J (2017). The fabrication of biomimetic biphasic CAN-PAC hydrogel with a seamless interfacial layer applied in osteochondral defect repair. Bone Res..

[16] Zhou C (2015). Tetraploid complementation proves pluripotency of induced pluripotent stem cells derived from adipose tissue. Cell Prolif..

[17] Johnstone B (2013). Tissue engineering for articular cartilage repair–the state of the art. Eur. Cell Mater..

[18] Huang H (2014). A functional biphasic biomaterial homing mesenchymal stem cells for *in vivo* cartilage regeneration. Biomaterials.

[19] Lee CH (2010). Regeneration of the articular surface of the rabbit synovial joint by cell homing: a proof of concept study. Lancet.

[20] Chen P (2015). Radially oriented collagen scaffold with SDF-1 promotes osteochondral repair by facilitating cell homing. Biomaterials.

[21] Ruel-Gariepy E, Leroux JC (2004). *In situ*-forming hydrogels–review of temperature-sensitive systems. Eur. J. Pharm. Biopharm..

[22] Billiet T, Vandenhaute M, Schelfhout J, Van VS, Dubruel P (2012). A review of trends and limitations in hydrogel-rapid prototyping for tissue engineering. Biomaterials.

[23] Richter A (2008). Review on Hydrogel-based pH Sensors and Microsensors. Sensors (Basel).

[24] Ahmed EM (2015). Hydrogel: Preparation, characterization, and applications: A review. J. Adv. Res..

[25] Liu CB (2008). Thermoreversible gel-sol behavior of biodegradable PCL-PEG-PCL triblock copolymer in aqueous solutions. J. Biomed. Mater. Res. B Appl. Biomater.

[26] Boffito M, Sirianni P, Di RA, Chiono V (2015). Thermosensitive block copolymer hydrogels based on poly(varepsilon-caprolactone) and polyethylene glycol for biomedical applications: state of the art and future perspectives. J. Biomed. Mater. Res. A.

[27] Ma G, Miao B, Song C (2010). Thermosensitive PCL-PEG-PCL hydrogels: Synthesis, characterization, and delivery of proteins. J. Appl. Polym. Sci..

[28] Jiang CP, Huang JR, Hsieh MF (2011). Fabrication of synthesized PCL‐PEG‐PCL tissue engineering scaffolds using an air pressure‐aided deposition system. Rapid Prototyp. J..

[29] Fu S (2010). Preparation and Characterization of Nano-Hydroxyapatite/Poly(ε-caprolactone)−Poly(ethylene glycol)−Poly(ε-caprolactone) Composite Fibers for Tissue Engineering. J. Phys. Chem. C.

[30] Ko CY (2016). *In vitro* and *in vivo* co-culture of chondrocytes and bone marrow stem cells in photocrosslinked PCL-PEG-PCL hydrogels enhances cartilage formation. J. Tissue Eng. Regen. Med..

[31] Fu N (2016). PCL-PEG-PCL film promotes cartilage regeneration *in vivo*. Cell Prolif..

[32] Pazarceviren E, Erdemli O, Keskin D, Tezcaner A (2017). Clinoptilolite/PCL-PEG-PCL composite scaffolds for bone tissue engineering applications. J. Biomater. Appl..

[33] Hou J (2016). Degradability, cytocompatibility, and osteogenesis of porous scaffolds of nanobredigite and PCL-PEG-PCL composite. Int. J. Nanomedicine.

[34] Gao X (2013). Novel thermosensitive hydrogel for preventing formation of abdominal adhesions. Int. J. Nanomedicine.

[35] Tran TQM (2016). Bactericidal Effect of Lauric Acid-Loaded PCL-PEG-PCL Nano-Sized Micelles on Skin Commensal Propionibacterium acnes. Polymers.

[36] Park JS (2007). *In vitro* and *in vivo* test of PEG/PCL-based hydrogel scaffold for cell delivery application. J. Control Release.

[37] Zhong J (2016). Crosstalk between adipose-derived stem cells and chondrocytes: when growth factors matter. Bone Res..

[38] Shi S (2016). Effects of low oxygen tension on gene profile of soluble growth factors in co-cultured adipose-derived stromal cells and chondrocytes. Cell Prolif..

[39] Gong T (2015). Nanomaterials and regenrative medicine. Bone Res..

[40] Liu L (2013). Camptothecine encapsulated composite drug delivery system for colorectal peritoneal carcinomatosis therapy: biodegradable microsphere in thermosensitive hydrogel. Colloids Surf. B Biointerfaces.

[41] Chu WS, Pandey JK, Ahn SH (2014). Fabrication of Bio-Composite Scaffold for Implantable Drug Delivery System (DDS). J. Biobased Mat. Bioenergy.

[42] Azouz L, Dahmoune F, Rezgui F, G’Sell C (2016). Full factorial design optimization of anti-inflammatory drug release by PCL-PEG-PCL microspheres. Mater. Sci. Eng. C Mater. Biol. Appl..

[43] Khodaverdi E (2015). Injectable supramolecular hydrogel from insulin-loaded triblock PCL-PEG-PCL copolymer and gamma-cyclodextrin with sustained-release property. AAPS PharmSciTech.

[44] Balakrishnan S (2016). Gold nanoparticle-conjugated quercetin inhibits epithelial-mesenchymal transition, angiogenesis and invasiveness via EGFR/VEGFR-2-mediated pathway in breast cancer. Cell Prolif..

[45] Wu H (2011). Chitosan-polycaprolactone copolymer microspheres for transforming growth factor-β1 delivery. Colloids Surf. B Biointerfaces.

[46] Yoshizawa H (2015). TGF-beta(1)-siRNA delivery with nanoparticles inhibits peritoneal fibrosis. Gene Ther..

[47] Li G (2015). Poly(3-hydroxybutyrate-co-4-hydroxybutyrate) Based Electrospun 3D Scaffolds for Delivery of Autogeneic Chondrocytes and Adipose-Derived Stem Cells: Evaluation of Cartilage Defects in Rabbit. J. Biomed. Nanotechnol..

[48] Gong CY (2009). Biodegradable *in situ* gel-forming controlled drug delivery system based on thermosensitive PCL-PEG-PCL hydrogel: part 1–Synthesis, characterization, and acute toxicity evaluation. J. Pharm. Sci..

[49] Cui Z (2012). Morphology and Properties of Porous and Interconnected Poly(ε-caprolactone) Matrices Using Solid and Microcellular Injection Molding. J. Biobased Mat. Bioenergy.

[50] Gong C (2009). Biodegradable *in situ* gel-forming controlled drug delivery system based on thermosensitive PCL-PEG-PCL hydrogel. Part 2: sol-gel-sol transition and drug delivery behavior. Acta Biomater..

[51] Chung JY (2014). Comparison of articular cartilage repair with different hydrogel-human umbilical cord blood-derived mesenchymal stem cell composites in a rat model. Stem Cell Res. Ther..

[52] Zhang T (2016). Softening Substrates Promote Chondrocytes Phenotype via RhoA/ROCK Pathway. ACS Appl. Mater. Interfaces.

